# Subclinical patterns of disordered eating behaviors in the daily life of adolescents and young adults from the general population

**DOI:** 10.1186/s13034-024-00752-w

**Published:** 2024-06-06

**Authors:** Stephanie K. V. Peschel, Christine Sigrist, Catharina Voss, Sophia Fürtjes, Johanna Berwanger, Theresa M. Ollmann, Hanna Kische, Frank Rückert, Julian Koenig, Katja Beesdo-Baum

**Affiliations:** 1 Behavioral Epidemiology, Institute of Clinical Psychology and Psychotherapy, TUD Dresden University of Technology, Dresden, Germany; 2 Faculty of Medicine, University of Bern, Bern, Switzerland; 3 Department of Child and Adolescent Psychiatry, Psychosomatics and Psychotherapy, Faculty of Medicine and University Hospital Cologne, University of Cologne, Cologne, Germany

**Keywords:** Disordered eating, Latent profile analysis, Ecological momentary assessment, Epidemiological study, Risk factors

## Abstract

**Background:**

Disordered eating behaviors (DEBs), a risk factor for the development of eating disorders (EDs), are prevalent in young people and different DEBs frequently co-occur. Previous studies on DEB-patterns have largely used traditional retrospective questionnaires to assess DEBs. In addition, most previous studies did not specifically exclude individuals with clinical EDs, which limits current knowledge concerning purely subclinical patterns of DEBs. In the present study, we aimed to explore phenotypes and group sizes of subclinical patterns of DEBs reported in everyday life via smartphone-based ecological momentary assessment (EMA) in adolescents and young adults from the general population without lifetime EDs. In secondary analyses, we further aimed to investigate whether DEB-patterns would be associated with additional previously identified risk factors for ED-development.

**Methods:**

EMA was conducted in a community sample of 14–21-year-olds from Dresden, Germany, over four days for up to eight times a day and covered engagement in four DEBs: skipping eating, restrained eating, eating large amounts of food, and loss-of-control eating. Data were analyzed from *N* = 966 individuals without lifetime EDs with an EMA compliance rate of at least 50% (81.9% of the total sample; average compliance: 84.6%). Latent profile analyses were performed to identify subclinical patterns of DEBs, stratified by sex. Associations between symptomatic profiles and ED-risk factors were tested via regression analyses.

**Results:**

Based on theoretical deliberations, statistical indices, interpretability, and parsimony, a three-profile solution, namely no DEBs, high-mixed DEBs, and low-mixed DEBs, was selected for both sexes. Both symptomatic profiles in both sexes were associated with more unfavorable manifestations in additional ED risk factors compared to the no DEBs profile, with the highest number of associations being observed in the female high-mixed profile.

**Conclusions:**

The present findings suggest that problematic manifestations of DEBs in young people may occur even in the absence of an ED diagnosis and that they are associated with additional risk factors for EDs, warranting increased efforts in targeted prevention, early identification and intervention in order to counteract symptom progression.

**Supplementary Information:**

The online version contains supplementary material available at 10.1186/s13034-024-00752-w.

## Background

Disordered eating behaviors (DEBs) refer to maladaptive eating behaviors, such as restrictive eating or binge eating, that represent symptoms of clinical eating disorders (EDs), but may also occur among individuals without clinical EDs with lesser intensity and/or frequency [1]. A recent meta-analysis reported that approximately 22% of children and adolescents are affected by problematic levels of ED symptoms [2]. Similarly, DEBs specifically are highly prevalent among both adolescents and young adults [3–5] and show a higher prevalence than clinical ED diagnoses [6, 7] For example, among 11–17 year old Germans, restrictive eating was affirmed by 34.8%, and engagement in binge eating was indicated by 16.0% of participants respectively [5]. A high prevalence of DEBs in young people is a cause for concern because they have been linked to numerous adverse psychosocial, physical, and mental health outcomes in both cross-sectional and longitudinal studies [7–12]. Critically, DEBs have also been shown to precede the onset of clinical eating disorders [13–17], which, once manifested, are related to detrimental health consequences, including increased mortality [18], as well as significant economic burden for the society [19]. Thus, young individuals engaging in subclinical DEBs represent a high-risk group for the development of clinical EDs that also most often have their onset in adolescence or young adulthood [20–22]. In this context, it is essential to study the phenotypes of specific DEBs as well as their co-occurrence with additional risk factors for ED development, such as eating-and body-related factors, aspects of psychological functioning and psychopathology, as well as facets of interpersonal difficulties [e.g., 14, 23–25], to inform prevention, detection, and early intervention.

It is important to note, that DEBs often do not manifest in an isolated manner. Rather, studies applying different cluster-analytical approaches in population- or community-based samples have identified several subgroups of DEB-patterns. These patterns have been characterized by different combinations and/or severities of specific DEBs, group sizes, as well as differential associations with adverse outcomes [26–33]. For example, based on five self-reported DEBs and closely related symptoms in 422 adolescent girls, Viborg et al. [33] identified six clusters. Those were described by the authors as (1) *no eating problems*, (2) *social eating problems*, (3) *fear of not being able to stop eating*, (4) *weight concerns*, (5) *multiple eating problems without purging*, and (6) *multiple eating problems including purging* [33]. Associations with adverse outcomes varied across the clusters. For example, the two multiple eating problems clusters showed greater self-reported psychological difficulties than all remaining clusters. Other clusters were also characterized by more unfavorable scores on adverse outcomes compared to the *no eating problems*-cluster, such as lower body esteem in all but the *social eating problem*-cluster [33].

Notably, the above-mentioned previous studies on different DEB-patterns in young people are marked by some methodological limitations. Firstly, previous cluster-analytical studies have typically either not reported clinical ED-diagnoses [e.g., 26, 29–31, 33] or included individuals both with and without diagnosed EDs [28]. Consequently, phenotypes of DEB patterns and their relationships with adverse outcomes/risk factors are less clear exclusively in young individuals without clinical lifetime EDs, who represent potential high-risk groups for developing these disorders. As a second limitation, previous cluster-analytical studies have largely relied on retrospective self-report questionnaires [e.g., 26, 28–33] that cover widely varying timeframes and are prone to recall-bias [34].

The use of ecological momentary assessment (EMA), which employs repeated (near-)real-time measurements of behaviors in everyday life and in the natural environment, resulting in reduced recall-bias and high ecological validity [35], could address the limitations of previous studies. EMA-data have been formerly used to identify different types of eating episodes that are shared across individuals, for example in women with Anorexia Nervosa (AN) [36] or regarding disinhibited eating in children and adolescents [37]. However, to the best of our knowledge, no previous study has followed this approach in the identification of distinct patterns of DEBs in young non-clinical people.

Accordingly, building on and extending the previous literature, the current study used available EMA-data collected in an epidemiological cohort study of adolescents and young adults to explore DEB-patterns based on four types of DEBs in both male and female individuals free from a lifetime ED-diagnosis. More specifically, the aim of the present study was to examine respective phenotypes, group sizes, and associations with additional risk factors for developing a clinical ED. Since to our knowledge no previous cluster-analytic study has examined subgroups based on the exact same set of DEBs included in the present study (i.e., restrained eating, skipping eating, loss-of-control eating, and eating large amounts of food), we refrained from making specific predictions regarding the number of subgroups or their respective sizes and instead followed a strictly exploratory approach. Nevertheless, based on previous findings, one large asymptomatic subgroup was expected, as well as several—to be specified—symptomatic subgroups [28–33]. Despite some inconsistent findings [32], given that some previous studies reported differences in DEB-patterns between males and females [26, 31, 38], the present work also examined DEB-patterns separated by sex.

To characterize subgroups, we focused on variables that met both of the following criteria: (1) Variables that have been implicated as risk factors for the development of EDs from a theoretical perspective. Specifically, we drew from a comprehensive systematic review on different theoretical models of disordered eating by Pennesi and Wade [24] in order to be able to include a broad thematic range of theoretical risk factors, rather than focusing on one specific isolated model. (2) Variables were further only included if there was at least some empirical evidence supporting their relevance as prospective risk factors for ED-development [14, 15, 23, 25, 39–44]. Specifically, we included facets of psychopathology, eating-and body-related aspects, aspects of psychological and interpersonal functioning, and developmental factors. We expected the symptomatic groups to be characterized by more unfavorable manifestations in risk factors compared to the asymptomatic group.

## Methods

### Study design and procedures

The present analyses are based on baseline data from the first cohort of the Behavior and Mind Health (BeMIND) study [45]. The study was conducted in line with the Declaration of Helsinki and ethical clearance was obtained from the ethics committee of TU Dresden (No. EK381102014). The BeMIND study was designed as a comprehensive cross-sectional and longitudinal epidemiological cohort study program investigating various aspects of health and disease of adolescents and young adults aged 14–21 years from the community living in the eastern German city of Dresden. All individuals in the respective age range living in Dresden at the times of sampling and data collection were generally eligible for participation. In 2015, a random age- and sex-stratified sample was drawn from the city’s municipal population registry and subsequently contacted via written mail (with a maximum of two reminder letters). Study participation required written informed consent/assent from the participants, as well as from all legal guardians in the case of minors. Exclusion criteria were insufficient German language skills, permanent institutionalization, and not living in Dresden during study conductance. Recruitment resulted in a total of *N* = 1,180 participants at baseline (participation/response rate: 21.7%). Most frequent reasons indicated for non-participation were lack of time and lack of interest. Further details on the recruitment process and baseline sample characteristics are provided in Beesdo-Baum et al. [45]. From 11/2015 to 12/2016, participants took part in the comprehensive baseline assessment, including diagnostic, experimental, and biomarker-related procedures. Two in-person appointments were conducted at the study center at TU Dresden, which took place approximately 7 days apart from each other. Both in-person appointments included the completion of self-report questionnaires on a tablet computer. In between the two appointments, participants completed EMA (please see below) and an online-assessment comprising various additional self-report questionnaires.

### Diagnostic interview

Within their first lab appointment, participants were interviewed face-to-face using an updated research version (DIA-X-5/D-CIDI) [46] of the computerized and fully standardized Munich Composite International Diagnostic Interview (DIA-X/M-CIDI) [47] to establish lifetime and 12-month diagnoses of mental disorders based on DSM-5 criteria [48]. ED-diagnoses covered by the DIA-X-5 are: AN, Bulimia Nervosa (BN), Binge-Eating Disorder (BED), other specified eating disorders (including atypical AN, purging disorder, BN of limited duration, and BED of limited duration), and unspecified eating disorders (UFEDs). Please note that BN and BED of limited frequency, respectively, were not specifically coded within the DIA-X-5/D-CIDI and affected individuals would fall into the UFED-category within the present study. Night Eating Syndrome and feeding disorders are not assessed within the DIA-X-5 and thus are not included. The DIA-X-5 has demonstrated good test-retest reliability for EDs [46]. Since the current study aimed to investigate strictly subclinical DEB-patterns and taking into account that individuals with past ED-diagnoses tend to show residual ED-symptoms even after remission or recovery [49], individuals with lifetime ED-diagnoses (dichotomously coded as presence or absence of any lifetime-ED or any lifetime other specified eating disorder or UFED) were excluded in the present analyses. Although UFEDs are less well defined than AN, BN, BED, and other specified eating disorders, individuals with UFEDs have previously shown levels of ED symptomatology comparable to those in other ED-diagnoses [50]. Thus, in the present study, UFED was considered a clinically relevant ED. When information on ED-diagnosis (*n* = 10) was missing, ED-diagnoses were conservatively coded as not fulfilled.

### Ecological momentary assessment

EMA-measurements took place over four consecutive days, including two week- and two weekend-days, and were implemented using a self-developed smartphone-app. Each day included a total of eight semi-randomly scheduled assessments (one assessment in the morning and before bedtime respectively, six assessments throughout the day) which were adjusted to individual sleeping and wake times, as well as time frames during which participants did not want to be disturbed. Participants were not aware of when exactly the assessments would be prompted and received an acoustic alarm whenever an assessment was to be completed. Assessments could be postponed three times for five min each or skipped entirely by the participant. The minimum time period between two consecutive assessments was 30 min. A small number of assessments (*n* = 18) occurring within less than 30 min (if the previous assessment had been postponed) were excluded from the analyses. Each EMA-assessment included overall 203–248 items, addressing a wide range of different experiences and behaviors in young people’s everyday lives. In order to facilitate efficient and quick assessments and to limit participant burden (resulting in ~ 3 min per assessment), branching rules were used (please see Beesdo-Baum et al. for details [45]. DEBs were measured at every assessment (see below).

Participants were equipped with a study smartphone and received a technical introduction from trained study personnel. On the day prior to the four-day EMA-period, participants were presented up to three practice assessments to allow for further familiarization with the procedure and to minimize reactivity. The practice-data are not included in the present analyses.

### Measures

#### Sociodemographic information

Self-reported social class was collected within the first in-person study appointment as part of questionnaires displayed on a tablet computer adjacent the personal diagnostic interview. Participants were asked to assign themselves to one out of five categories: lowest, lower middle, middle, upper middle, or upper class. The level of education was judged by information provided during the demographic section of the diagnostic interview. Participants were initially asked to provide information on whether they were still attending school. If they affirmed, the type of primary/secondary school they attended was assessed. In those indicating that they had completed school, the type of primary/secondary school degree that they had obtained was collected. Based on this information, individuals were allocated to one out of four categories of education: low (corresponding to elementary school or lower secondary school), middle (corresponding to secondary school), high (corresponding to higher secondary school/A-level), and other (corresponding to, e.g., private schools).”

#### Disordered eating behaviors

Self-reported severities of four distinct DEBs were assessed throughout the EMA period, using one single item each after initially asking participants whether they had eaten since the last assessment. If participants stated they had not, one item assessing *skipping eating* (“You have stated you have not eaten since the last assessment. Was one reason not to eat the attempt to control your weight or body shape?”) was presented. If eating was affirmed, *restrained eating* (“Since the last assessment, I tried to limit the amount of food that I ate”), *loss-of-control eating* (“Since the last assessment, I lost control over my eating behavior), and *eating large amounts of food* (“Since the last assessment, I ate as if in a rush or I ate a large amount of food given the circumstances”) were assessed. All items were rated on a continuous seek bar, ranging from *not at all* (0) to *absolutely* (100), with higher values indicating greater DEB severity. Items were adapted from another study [51] in which items had been adapted for EMA-purposes from the well-established Eating Disorder Examination-Questionnaire [52]. Importantly, while it is acknowledged that a variety of other DEBs, such a dysfunctional compensatory behaviors, exist [e.g., 5], the BeMIND study did not focus on eating pathology, but rather aimed to assess a large variety of aspects of mental health. Given the need to limit participant burden, we only assessed four types of DEBs within the present study. Related limitations are addressed in the discussion. Within a previous study [53] also based on the baseline assessment of the first BeMIND study cohort, we were able to demonstrate that, individuals with 12-month ED-diagnoses were characterized by significantly higher average levels across the four types of DEBs compared to participants without, which provides tentative support for the items’ validity.

#### Psychopathology

Self-reported *depressive symptoms* within the past 2 weeks were measured using the German version of the depression module of the Patient Health Questionnaire (PHQ-9) [54, 55] which was administered during the first lab appointment on a tablet computer. A maximum of two missing items are allowed to calculate the total score. The total score (possible range: 0–27, with higher scores reflecting greater symptom severity) is obtained by summing the scores across all available items, multiplying it by 9, and dividing it by the number of items completed. Cronbach’s alpha within the present sample was 0.79.

Self-reported *anxiety symptoms* within the past 4 weeks were measured using the German version of the Cross-Cutting Dimensional Anxiety Scale (Cross-D) [56, 57] displayed on a tablet computer during the first appointment. A higher sum score (possible range: 0–40) across all ten items indicates greater symptom severity. Cronbach’s alpha within the present sample was 0.83.

#### Eating-and body-related factors

*Self-reported engagement in dieting within the past 12 months* was measured as part of the online assessment using the following single item adapted from based on the Health Behaviour in School-aged Children study [58, 59]: “How often have you gone on a diet within the past 12 months? By ‘diet’ we mean changing one’s eating habits so one can lose weight.” Participants selected their reply from 5 categories (0 = *no time*, 4 = *seven times or more*). For the present analyses, in line with previous work [4], the item was dichotomously coded (no dieting vs. any dieting within the past 12 months).

*Presence of lifetime weight-, shape-, and eating-concerns* was assessed using a single item administered within the diagnostic interview (“Was there ever a time in your life when you had a great deal of concern about your weight, how much you eat, or being too fat?”; dichotomously coded as *yes* or *no*).

*Self-reported body satisfaction* was measured within the online assessment using a single item (“How satisfied are you with your overall physical appearance?”) which was adapted from Merikangas et al. [60]. A German adaptation was developed by the BeMIND-study team. Body satisfaction was rated on an 11-point Likert scale (0 = *not satisfied at all* to 10 = *very satisfied*).

The *eating as a means of coping with emotional stress-*subscale from the Eating Behaviour and Weight Problems Inventory for Children (EWI-C) [61], completed during the second in-person appointment on a tablet computer, was used as an approximate measure of emotional eating. A higher subscale-score (mean across eight items rated from 0 to 3 multiplied by ten; possible range: 0–30) indicates a greater extent to which a person reacts with (increased) food intake to emotional distress. Cronbach’s alpha within the present sample was 0.86.

*Attitude toward the obese* (considered as an approximation of the *thin ideal* previously described as an ED-risk factor [14]) were also measured using the respective subscale from the EWI-C [61]. A higher subscale-score across five items (mean across five items rated from 0 to 3 multiplied by ten; possible range: 0–30) indicates more negative attitudes towards higher weight people. Cronbach’s alpha within the present sample was 0.82.

#### Psychological functioning

The Short Scale for Measuring General Self-efficacy Beliefs (ASKU) [62] was delivered as a self-report measure for *self-efficacy* within the online assessment. A mean score (possible range: 1–5) is calculated based on three items, with a higher score indicating higher self-efficacy. Cronbach’s alpha within the present sample was 0.86.

*Self-esteem* was measured using the Single-Item Self-Esteem Scale (SISE) [63] within the online assessment. A German translation was developed by the BeMIND-study team. A higher rating (range: 1–7) indicates a higher self-esteem.

To assess *trait emotion regulation skills*, participants filled out the Emotion Regulation Skills Questionnaire (ERSQ) [64] comprising 27 items as part of the online assessment. The mean across all items represents the total ERSQ score (possible range: 0–4), with higher scores corresponding to a more frequent implementation of adaptive emotion regulation skills. Cronbach’s alpha within the present sample was 0.94.

#### Interpersonal factors

As an approximate measure of *interpersonal functioning*, which has been identified as a prospective ED risk factor [14], the Relationship Questionnaire (RQ) [65, 66] assessing self-reported adult attachment styles was administered within the online assessment. The RQ comprises four items, relating to one out of four adult attachment styles, respectively (secure, preoccupied, dismissing, and fearful), each depicting a short description of the concept of the self and others in the context relationships. A higher score (possible range: 1–7) indicates greater endorsement of the respective attachment style. Since secure attachment is not expected to be a risk factor for the development of EDs, only ratings of the three remaining styles were included as characterizing variables in the present study.

*Self-reported social support* was measured using the Oslo-3-Items-Social-Support Scale [67] administered within the online questionnaire assessment. Items are rated on a scale from 1 to 4 (item 1) and 1–5 (items 2 and 3) and are summed for a total score (possible range: 3–14), with higher scores indicating greater perceived social support. Wording of item three was slightly adapted for a better suitability towards the age group in the present sample. Cronbach’s alpha within the present sample was 0.63.

#### Developmental factors

Experiences of *sexual abuse* and *physical neglect* in childhood and/or adolescence were measured via the respective subscales of the German version [68] of the Childhood Trauma Questionnaire (CTQ) [69] administered on a tablet computer within the first in-person appointment. Each subscale contains five items that are summed for a subscale score respectively, with higher scores (possible range: 5–25) indicating more severe related experiences. Cronbach’s alpha within the present sample was 0.85 for sexual abuse and 0.39 for physical neglect. Low internal consistency for the physical neglect subscale, in contrast to other CTQ-subscales, has been previously reported in other studies [68, 70] and caution when interpreting this outcome is warranted.

As a measure of perceived *adverse parental behaviors* (as approximations of previous adverse aspects of parental behaviors/relationships with parents identified as prospective ED-risk factors [42, 43]), participants completed the German version [71] of the Measure of Parental Style (MOPS) [72] within the online assessment. The MOPS contains three subscales, each assessed separately for perceived maternal and paternal behavior respectively. In line with the German version of the questionnaire, mean scores were obtained across the respective items rated from 1 to 3: indifference (6 items), abuse (6 items), and over-control (3 items). In the German version [71], and in contrast to the original version [72], one item has been moved from the over-control-subscale to the abuse-subscale due to higher factors loadings. The MOPS for maternal or paternal behaviors was only completed if participants indicated that they were raised by a female or male caregiver, respectively. Otherwise, respective scores are missing. Cronbach’s alphas within the present sample were as follows: maternal indifference 0.90, paternal indifference 0.93, maternal abuse 0.85, paternal abuse 0.90, maternal over-control 0.64, and paternal over-control 0.62.

#### Body Mass Index standard deviation score

The Body Mass Index Standard Deviation Score (BMI-SDS; i.e., the individual standard deviation of an individual’s BMI (kg/m^2^) from the age- and sex-specific BMI-median) was calculated based on weight and height of the participants. These measurements were typically obtained during the second lab visit using a standard digital scale and stadiometer. In case of missing values, self-reported height and weight from the ED-section of the DIA-X-5 were used. BMI-SDS was calculated using the method proposed by Cole et al. [73], based on national BMI norms [74, 75].

### Data analytic plan

#### Inclusion and exclusion of participants

From the initial total sample (*N* = 1,180), *n* = 111 (9.4%) were excluded due to a lifetime ED-diagnosis or other specified eating disorder/UFED-diagnosis. Further, *n* = 103 were excluded from the present analyses due to the following methodological reasons: *n* = 25 did not complete any EMA, *n* = 74 filled in less than 50% of EMA-assessments resulting in unreliable and insufficient data, *n* = 3 provided implausible information on eating behavior (i.e., did not indicate any eating episode during any of the EMA-assessments), and *n* = 1 indicated eating during every assessment, therefore lacking values for the subsequent question on skipping eating and thus the respective indicator variable for latent profile analysis (LPA).

The final sample consisted of *N* = 966 (81.9% of the total sample, *n* = 537 female, *n* = 429 male).

#### Statistical analyses

LPAs separated by sex were performed in *RStudio* [76] (Version 2023.3.0.386, R version 4.3.0.) using the Package *TidyLPA* (Version 1.1.0) [77]. Participants’ average scores of each DEB, based on available EMA reports, were used as indicator variables to fit models ranging from one to six profiles. In line with current standards and recommendations, theoretical deliberations, interpretability, parsimony, and different statistical measures [78–80] were considered to inform model selection (i.e., to select an adequate profile solution). With respect to statistical indices, we first considered the Bayesian information criterion (BIC) [81] and the sample size-adjusted BIC (SABIC) [82], with lower values indicating better model fit. The BIC and SABIC have demonstrated good performance in selecting the appropriate number of profiles [83]. Critically, the distribution of our indicator variables was right-skewed (with most individuals reporting low average levels of DEBs), which has been shown to have a potentially detrimental effect on model selection based on the BIC and SABIC [79]. Accordingly, we also considered entropy [84], which has been shown to be less affected by non-normality of indicators [79]. Entropy [84] indicates the degree of certainty with which individuals are assigned to profiles. Higher values (range: 0–1) indicate a better fit and should ideally be 0.8 or higher [78, 80]. Finally, we used the bootstrapped likelihood ratio test (BLRT) [85] to determine whether a solution with *n* profiles would provide an advantage over *n* − 1 profiles, as indicated by a significant test result. Individuals were each assigned to their most likely profile based on posterior probabilities [77]. Given the exploratory nature of the present LPA, we initially conducted all analyses with two different model specifications: (a) assuming equal variances and equal covariances, and (b) assuming varying variances and varying covariances. Since the former specifications consistently yielded poor entropy values (< 0.55), the specifications listed in (b) were retained.

All other analyses were conducted in Stata 17 [86]. Following standard procedures [78], subsequent to LPA, we investigated whether symptomatic profiles would be associated with more unfavorable manifestations of correlates previously identified as ED-risk factors within other studies compared to the asymptomatic group. Separated by sex, we performed linear and logistic regressions with the profile-membership as the independent variable and the asymptomatic profile set as the base group, and correlates as dependent variables. Continuous correlates were *z*-standardized so that beta-coefficients comparing profiles could be interpreted as Cohen’s *d*, with values ranging from 0.2 to < 0.5 indicating a small effect size, values from 0.5 to < 0.8 indicating a medium effect size, and values > 0.8 indicating a large effect size [87]. All regression models were adjusted for age. Missing values for correlate-variables were handled by pairwise deletion. Information on missing values for each dependent variable can be retrieved in the Supplementary Material (Tables S1 and S2). For descriptive purposes, we also tested whether profiles differed by age and BMI-SDS, which was tested by linear regression. BMI-SDS was not *z*-standardized since its units already indicate standard deviations. Age was also not *z*-standardized to facilitate interpretation.

We applied a conventional level of significance of *p* < 0.05. Because of the exploratory nature of the present analyses, we did not correct for multiple testing [88]. Therefore, caution is warranted in the interpretation of significant results. Further, although originally intended for all analyses pertaining to the BeMIND study [45], analyses were not weighted regarding age and sex because the *TidyLPA*-package, to the best of our knowledge, does not support sampling weights. Related implications are discussed in the limitations-section.

## Results

### Sample characteristics

Sociodemographic information and descriptive statistics separated by sex are provided in Table 1. Differences in sociodemographic characteristics were tested comparing the included sample to both the subsample excluded due to lifetime EDs, as well as those excluded due to methodological reasons, respectively. More information can be retrieved in the Supplementary Material (S3). In brief, the excluded subsample with a lifetime ED was older (*p* < 0.001) had a higher proportion of females (*p* < 0.001), and a higher BMI-SDS (*p* = 0.002) compared to the included participants, but there were no differences regarding social class, education, and nationality. Participants excluded due to methodological reasons were significantly different from the included ones regarding education (*p* < 0.001), with a higher proportion of individuals with low education (*p* < 0.001), and a lower proportion of individuals with high education (*p* = 0.001) compared to the included sample. There were no differences regarding age, sex, social class, nationality, and BMI-SDS. Information on the exclusion of single EMA-assessments can be retrieved in the Supplementary Material (S4).

**Table 1:** Sample descriptives of the analysis sample separated by sex

Variables	Females *n* = 537	Males *n* = 429
Age, mean (SD)	17.23 (2.28)	17.15 (2.28)
German nationality, n (%)	528 (98.3)	414 (96.5)
Education, n (%)		
Low, n (%)	5 (0.9)	8 (1.9)
Middle, n (%)	100 (18.6)	83 (19.4)
High, n (%)	415 (77.3)	325 (75.8)
Other, n (%)	17 (3.2)	13 (3.0)
Social class, n (%)^a^		
Lowest, n (%)	9 (1.7)	9 (2.1)
Lower middle, n (%)	67 (12.5)	53 (12.4)
Middle, n (%)	329 (61.3)	259 (60.4)
Upper middle, n (%)	120 (22.4)	97 (22.6)
Upper, n (%)	3 (0.6)	2 (0.5)
Severities of DEBs (possible range: 0–100)		
Skipping eating, mean (SD)	5.13 (10.43)	4.19 (8.50)
Min– Max	0–91.67	0–56.16
Eating large amounts of food, mean (SD)	4.79 (7.75)	5.25 (8.53)
Min– Max	0–56.71	0–54.93
Loss-of-control eating, mean (SD)	4.76 (8.14)	4.87 (9.55)
Min– Max	0–76.80	0–100
Restriction, mean (SD)	10.46 (16.01)	8.30 (14.00)
Min– Max	0–99.75	0–89
BMI-SDS, mean (SD)	0.15 (0.88)	0.03 (0.99)
EMA-compliance in %, mean	85.2 (12.06)	83.7 (13.70)

BMI-SDS, Body Mass Index standard deviation score; EMA, ecological momentary assessment

^a^available from *n* = 528 in females, *n* = 420 in males

### Latent profile analyses

**Fig. 1:**
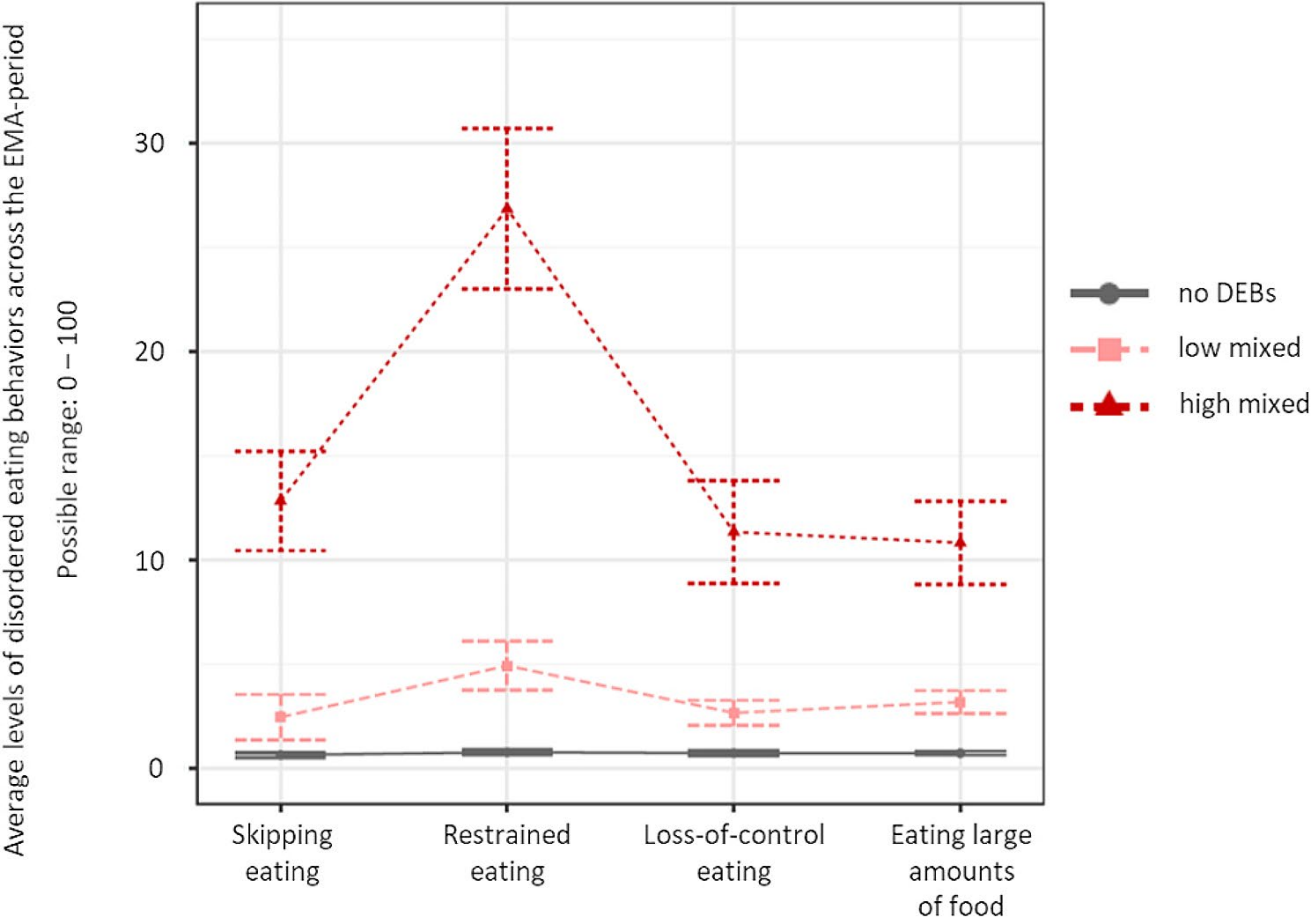
Profile plot for the final three-profile solution in females. *Note* Profile centroids and 95% confidence intervals. DEBs, disordered eating behaviors; EMA, ecological momentary assessment

**Fig. 2:**
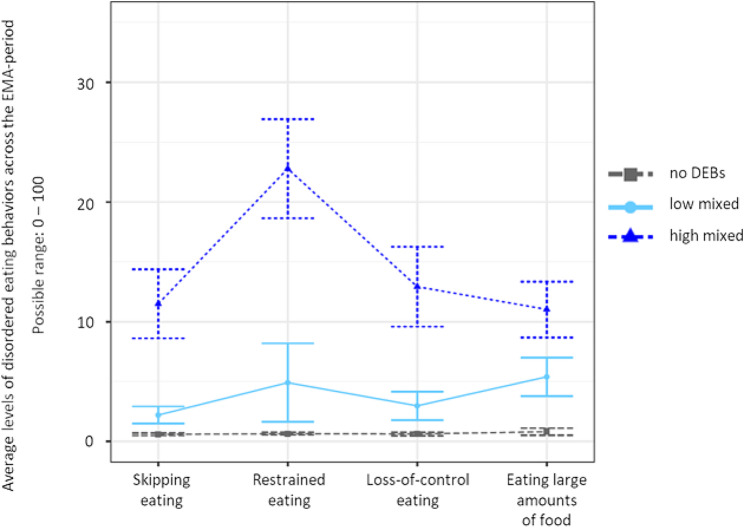
Profile plot for the final three-profile solution in males. *Note* Profile centroids and 95% confidence intervals. DEBs, disordered eating behaviors; EMA, ecological momentary assessment

Statistical indices for all potential model solutions inspected in both females and males are displayed in Table 2. For both females and males, the BLRT continued to yield a significant result with every additional profile, which is a familiar phenomenon [78]. Similarly, entropy was high across profile solutions, and BIC/SABIC kept decreasing with adding each additional profile. For both males and females, however, the inspection of scree plots (see the Supplementary Material, Figures S5 and S6) displaying BICs for each profile solution indicated a sharp decrease for the three-profile-model, with only small decreases as more profiles were added. This indicated a good statistical fit for the three-profile-solutions. In terms of interpretability, it was concluded that the four-profile solutions were not superior to the three-profile solutions. Considering the characteristics of the BIC as well as interpretability and parsimony, the three-profile solutions were retained for both females and males respectively. For the sake of transparency, profile plots for the four-and five-profile solutions in females and males respectively are provided in the Supplementary Material (Figures S7–S10). Profile plots separated by sex for the final model solution are displayed in Figs. 1 and 2. Profiles for both sexes were named no DEBs (females *n* = 173, 32.2%; males *n* = 160; 37.3%), high-mixed DEBs (females *n* = 168, 31.3%; males *n* = 117; 27.3%), and low-mixed DEBs (females *n* = 196, 36.5%; males *n* = 152, 35.4%). Within the profiles labeled as “no DEBs”, values for all DEBs were close to zero. Within the profiles labeled as “high-mixed DEBs”, levels of all four DEBs were markedly elevated, with restrained eating being the descriptively most highly elevated DEB compared to the three remaining ones. Finally, the profiles labeled as “low-mixed DEBs” was characterized by only mildly elevated levels of all four DEBs.

**Table 2:** Fit indices for 1–6 profile solutions in females and males

Group	Profiles	BIC	SABIC	Entropy	BLRT value	BLRT *p*-value
Females	1	15,388	15,344	1	NA	NA
	2	12,286	12,194	0.98	3197	0.01
	3^a^	11,401	11,262	0.94	979	0.01
	4	11,240	11,052	0.90	256	0.01
	5	11,094	10,859	0.90	240	0.01
	6	10,972	10,689	0.89	217	0.01
Males	1	12,541	12,496	1	NA	NA
	2	9680	9588	0.98	2952	0.01
	3^a^	9044	8904	0.93	727	0.01
	4	8877	8690	0.91	257	0.01
	5	8748	8513	0.91	220	0.01
	6	8594	8311	0.90	245	0.01

BIC, Bayesian information criterion; BLRT, bootstrapped likelihood ratio test; NA, not applicable; SABIC, sample size-adjusted Bayesian information criterion

^a^Selected profile solution

### Validation analyses females

Descriptive values for validation variables separated by profile and statistics for regression analyses are provided in Table 3. Compared to the no DEBs-profile, individuals in the low-mixed and high-mixed profiles had higher depressive symptom scores, lower body satisfaction, lower social support, and higher physical neglect scores. Further, the low-mixed profile showed higher scores for maternal indifference. Additionally, the high-mixed profile showed higher anxiety symptom scores, greater odds of both dieting within the past year and lifetime weight-, shape- and eating concerns, higher scores for eating as a means of coping with emotional stress, lower emotion regulation skills, higher scores for both fearful and dismissing attachment, as well as higher scores for maternal over-control and abuse, and paternal indifference compared to the no DEBs-profile. Effect sizes were mostly small. Both symptomatic profiles did not significantly differ from the no DEBs profile with respect to age (*p*’s > 0.20). However, BMI-SDS was higher in the low-mixed (*M* = 0.16, *SD* = 0.81, *b* = 0.30, CI 95% 0.12–0.47, *p* = 0.001) and in the high-mixed profile (*M* = 0.43, *SD* = 0.87, *b* = 0.57, CI 95% 0.39–0.75, *p* < 0.001) compared to the no DEBs profile (*M* = − 0.14, *SD* = 0.88).

### Validation analyses males

Descriptive values for validation variables separated by profile and statistics for regression analyses are provided in Table 4. Both the high and low-mixed profiles had significantly greater odds of dieting in the past year and higher scores in preoccupied attachment compared to the no DEBs profile. Please note the wide confidence intervals pertaining to the effect of dieting, likely due to the overall small number of cases confirming dieting. Additionally, compared to the no DEBs-profile, the high-mixed profile had significantly greater odds of lifetime weight-, shape and eating concerns, lower body satisfaction, higher physical neglect scores, and higher scores for maternal/paternal abuse and indifference, as well as paternal over-control. Again, effect sizes were mostly small.

Compared to the no DEBs-profile (*M* = − 0.24, *SD* = 0.85), BMI-SDS was higher both in the low-mixed (*M* = 0.06, *SD* = 1.02, *b* = 0.30, CI 95% 0.09–0.52, *p* = 0.006) and in the high-mixed profile (*M* = 0.37, *SD* = 1.03, *b* = 0.62, CI 95% 0.39–0.52, *p* < 0.001). Finally, the participants in the high-mixed profile were younger than participants in the no DEBs-profile (*b* = − 0.62, CI 95% − 1.17 to − 0.08, *p* = 0.024), while age did not differ between the low-mixed profile and the no DEBs-profile (*p* > 0.40).

**Table 3:** Descriptive values in correlates (putative risk factors) separated by profile and regression analyses comparing the symptomatic with the asymptomatic profiles: Females

Correlates	No DEBs *n* = 173		Low-mixed *n* = 196		High-mixed *n* = 168		Low-mixed vs. no DEBs					High-mixed vs. no DEBs				
	*M*/(*n*)	(*SD*)/%	*M*/(*n*)	(*SD*)/%	*M*/(*n*)	(*SD*)/%	β/(OR)	*SE*	95% CI		*p*	β/(OR)	*SE*	95% CI		*p*
Psychopathology																
Depressive symptoms (PHQ-9)	3.73	(3.21)	4.57	(3.67)	5.55	(4.31)	0.21	0.10	0.01	0.41	**0.043**	0.48	0.11	0.27	0.69	**< 0.001**
Anxiety symptoms (CROSS-D)	5.09	(4.71)	6.37	(5.60)	7.61	(6.51)	0.20	0.10	− 0.004	0.41	0.055	0.45	0.11	0.24	0.66	**< 0.001**
Eating-and body-related factors																
Dieting within the past 12 months^a^	(16)	9.9	(26)	13.8	(45)	28.3	(1.44)	0.49	0.74	2.80	0.280	(3.65)	1.16	1.96	6.80	**< 0.001**
Eating as a means of coping with emotional stress (EWI-C)	4.47	(6.45)	5.14	(6.53)	6.71	(7.08)	0.09	0.10	− 0.12	0.29	0.403	0.34	0.11	0.13	0.55	**0.002**
Attitude toward the obese (EWI-C)	6.01	(6.28)	7.10	(7.10)	6.02	(6.48)	0.14	0.10	− 0.06	0.34	0.182	0.01	0.11	− 0.20	0.22	0.905
Lifetime weight-, shape and eating concerns^a^	(42)	24.30	(65)	33.2	(82)	49.1	(1.54)	0.36	0.98	2.44	0.063	(3.01)	0.71	1.90	4.78	**< 0.001**
Body satisfaction	7.01	(1.85)	6.36	(1.99)	6.01	(2.11)	− 0.32	0.11	− 0.53	− 0.11	**0.003**	− 0.50	0.11	− 0.72	− 0.28	**< 0.001**
Psychological functioning																
Self-esteem (SISE)	4.82	(1.48)	4.66	(1.50)	4.53	(1.70)	− 0.10	0.11	− 0.31	0.12	0.370	− 0.19	0.11	− 0.41	0.03	0.093
Self-efficacy (ASKU)	3.86	(0.60)	3.80	(0.61)	3.72	(0.70)	− 0.11	0.11	− 0.32	0.10	0.310	− 0.22	0.11	− 0.44	0.003	0.053
Emotion regulation skills (ERSQ)	2.71	(0.59)	2.63	(0.61)	2.53	(0.56)	− 0.14	0.11	− 0.35	0.07	0.200	− 0.31	0.11	− 0.53	− 0.09	**0.007**
Interpersonal factors																
Social support (OSLO)	11.70	(1.64)	11.30	(1.80)	10.75	(1.97)	− 0.23	0.11	− 0.44	− 0.02	**0.033**	− 0.51	0.11	− 0.72	− 0.29	**< 0.001**
Preoccupied attachment (RQ)	2.99	(1.81)	3.32	(1.90)	3.31	(1.78)	0.19	0.11	− 0.02	0.40	0.081	0.17	0.11	− 0.05	0.39	0.129
Fearful attachment (RQ)	3.01	(1.89)	3.37	(2.02)	3.68	(2.03)	0.18	0.11	− 0.03	0.40	0.091	0.34	0.11	0.12	0.56	**0.003**
Dismissing attachment (RQ)	3.65	(1.82)	3.84	(1.73)	4.07	(1.82)	0.12	0.11	− 0.09	0.33	0.275	0.22	0.11	0.004	0.45	**0.046**
Developmental factors																
Physical neglect (CTQ)^b^	5.71	(1.47)	6.23	(2.16)	6.16	(1.94)	0.27	0.10	0.06	0.47	**0.011**	0.24	0.11	0.03	0.45	**0.027**
Sexual abuse (CTQ)	5.10	(0.60)	5.36	(1.88)	5.10	(0.54)	0.20	0.10	− 0.005	0.41	0.055	0.001	0.11	− 0.21	0.21	0.994
Maternal overcontrol (MOPS)	1.58	(0.60)	1.63	(0.61)	1.73	(0.63)	0.09	0.11	− 0.12	0.31	0.404	0.25	0.11	0.03	0.47	**0.028**
Maternal abuse (MOPS)	1.26	(0.46)	1.31	(0.49)	1.37	(0.52)	0.09	0.11	− 0.12	0.31	0.401	0.22	0.11	0.001	0.45	**0.049**
Maternal indifference (MOPS)	1.15	(0.40)	1.24	(0.50)	1.22	(0.40)	0.22	0.11	0.004	0.44	**0.046**	0.16	0.11	− 0.06	0.38	0.163
Paternal overcontrol (MOPS)	1.34	(0.51)	1.38	(0.52)	1.46	(0.60)	0.09	0.11	− 0.13	0.31	0.430	0.21	0.12	− 0.02	0.44	0.070
Paternal abuse (MOPS)	1.22	(0.51)	1.35	(0.65)	1.34	(0.56)	0.21	0.11	− 0.01	0.43	0.063	0.21	0.12	− 0.02	0.44	0.074
Paternal indifference (MOPS)	1.19	(0.49)	1.30	(0.64)	1.33	(0.59)	0.18	0.11	− 0.04	0.40	0.108	0.24	0.12	0.01	0.47	**0.037**

Descriptives are reported unstandardized. Regression analyses were carried out based on z-standardized dependent variables and adjusted for age. *p*-values < 0.05 are printed in bold

ASKU, Short Scale for Measuring General Self-Efficacy Beliefs; BMI-SDS, Body Mass Index standard deviation score; CROSS-D, Cross-Cutting Dimensional Anxiety Scale; CTQ, Childhood Trauma Questionnaire; DEBs, disordered eating behaviors; ERSQ, Emotion Regulation Skills Questionnaire; EWI-C, Eating Behaviour and Weight Problems Inventory for Children; MOPS, Measure of Parental Style; N, Number; OSLO, OSLO-3-Item-Social-Support Scale; PHQ-9, Patient Health Questionnaire; RQ, Relationship Questionnaire; SISE, Single-Item Self-Esteem Scale

^a^Please note that this construct is coded dichotomously

^b^Please carefully consider the low Cronbach’s alpha (0.39) of this subscale in the present study sample (similar to some previous studies [68, 70]) when interpreting respective results

**Table 4:** Descriptive values in correlates (putative risk factors) separated by profile and regression analyses comparing the symptomatic with the asymptomatic profiles: Males

Correlates	No DEBs *n* = 160		Low-mixed *n* = 152		High-mixed *n* = 117		Low-mixed vs. no DEBs					High-mixed vs. no DEBs				
	*M*/(*n*)	(*SD*)/%	*M*/(*n*)	(*SD*)/%	*M*/(*n*)	(*SD*)/%	β/(OR)	*SE*	95% CI		*p*	β/(OR)	*SE*	95% CI		*p*
Psychopathology																
Depressive symptoms (PHQ-9)	3.40	(2.86)	3.82	(3.14)	3.55	(2.79)	0.13	0.11	− 0.09	0.36	0.236	0.08	0.12	− 0.16	0.32	0.509
Anxiety symptoms (CROSS-D)	3.94	(4.28)	4.82	(5.07)	4.25	(3.28)	0.19	0.11	− 0.03	0.41	0.087	0.11	0.12	− 0.13	0.35	0.367
Eating-and body-related factors																
Dieting within the past 12 months^a^	(3)	2.0	(12)	8.8	(12)	11.2	(4.78)	3.15	1.32	17.37	**0.017**	(6.80)	4.52	1.85	24.99	**0.004**
Eating as a means of coping with emotional stress (EWI-C)	2.43	(4.33)	2.69	(3.83)	3.12	(4.50)	0.05	0.12	− 0.17	0.28	0.636	0.19	0.12	− 0.05	0.43	0.127
Attitude toward the obese (EWI-C)	8.60	(8.08)	9.83	(7.50)	9.18	(7.82)	0.15	0.12	− 0.08	0.38	0.191	0.10	0.12	− 0.15	0.34	0.439
Lifetime weight-, shape and eating concerns^a^	(17)	10.6	(28)	18.5	(30)	25.6	(1.89)	0.63	0.99	3.63	0.054	(3.05)	1.02	1.58	5.89	**0.001**
Body satisfaction	6.82	(1.92)	6.39	(1.80)	6.13	(2.06)	− 0.22	0.12	− 0.46	0.02	0.069	− 0.35	0.13	− 0.61	− 0.09	**0.007**
Psychological functioning																
Self-esteem (SISE)	5.05	(1.49)	4.99	(1.34)	4.97	(1.49)	− 0.04	0.12	− 0.28	0.19	0.717	− 0.05	0.13	− 0.31	0.20	0.671
Self-efficacy (ASKU)	3.97	(0.73)	3.93	(0.69)	3.80	(0.74)	− 0.06	0.12	− 0.29	0.17	0.613	− 0.21	0.13	− 0.46	0.04	0.105
Emotion regulation skills (ERSQ)	2.67	(0.61)	2.56	(0.58)	2.58	(0.68)	− 0.16	0.12	− 0.40	0.07	0.172	− 0.14	0.13	− 0.39	0.12	0.282
Interpersonal factors																
Social support (OSLO)	10.67	(1.90)	10.60	(1.64)	10.53	(1.78)	− 0.04	0.12	− 0.28	0.20	0.727	− 0.08	0.13	− 0.33	0.18	0.550
Preoccupied attachment (RQ)	3.05	(1.65)	3.68	(1.73)	3.75	(1.56)	0.37	0.12	0.14	0.61	**0.002**	0.40	0.13	0.15	0.66	**0.002**
Fearful attachment (RQ)	3.31	(1.77)	3.59	(1.77)	3.64	(1.69)	0.16	0.12	− 0.07	0.40	0.177	0.21	0.13	− 0.04	0.47	0.100
Dismissing attachment (RQ)	4.48	(1.55)	4.28	(1.70)	4.42	(1.57)	− 0.12	0.12	− 0.36	0.11	0.304	− 0.07	0.13	− 0.32	0.19	0.616
Developmental factors																
Physical neglect (CTQ)^b^	6.01	(1.74)	6.26	(1.95)	6.41	(2.14)	0.12	0.11	− 0.10	0.34	0.283	0.25	0.12	0.01	0.49	**0.044**
Sexual abuse (CTQ)	5.09	(0.60)	5.05	(0.29)	5.08	(0.51)	− 0.09	0.11	− 0.32	0.13	0.409	0.01	0.12	− 0.23	0.25	0.920
Maternal overcontrol (MOPS)	1.72	(0.67)	1.77	(0.58)	1.89	(0.71)	0.07	0.12	− 0.17	0.30	0.589	0.26	0.13	− 0.001	0.51	0.051
Maternal abuse (MOPS)	1.29	(0.47)	1.32	(0.48)	1.49	(0.61)	0.05	0.12	− 0.18	0.29	0.649	0.35	0.13	0.10	0.61	**0.007**
Maternal indifference (MOPS)	1.18	(0.48)	1.25	(0.58)	1.38	(0.70)	0.11	0.12	− 0.12	0.35	0.347	0.33	0.13	0.07	0.58	**0.013**
Paternal overcontrol (MOPS)	1.39	(0.47)	1.50	(0.53)	1.68	(0.80)	0.19	0.12	− 0.05	0.43	0.115	0.46	0.13	0.20	0.72	**0.001**
Paternal abuse (MOPS)	1.24	(0.41)	1.39	(0.57)	1.53	(0.82)	0.24	0.12	− 0.003	0.48	0.052	0.45	0.13	0.19	0.71	**0.001**
Paternal indifference (MOPS)	1.17	(0.40)	1.31	(0.63)	1.48	(0.84)	0.22	0.12	− 0.02	0.46	0.075	0.46	0.13	0.20	0.72	**0.001**

Descriptives are reported unstandardized. Regression analyses were carried out based on z-standardized dependent variables and adjusted for age. *p*-values < 0.05 are printed in bold

ASKU, Short Scale for Measuring General Self-Efficacy Beliefs; BMI-SDS, Body Mass Index standard deviation score; CROSS-D, Cross-Cutting Dimensional Anxiety Scale; CTQ, Childhood Trauma Questionnaire; DEBs, disordered eating behaviors; ERSQ, Emotion Regulation Skills Questionnaire; EWI-C, Eating Behaviour and Weight Problems Inventory for Children; MOPS, Measure of Parental Style; N, number; OSLO, OSLO-3-Item-Social-Support Scale; PHQ-9, Patient Health Questionnaire; RQ, Relationship Questionnaire; SISE, Single-Item Self-Esteem Scale

^a^Please note that this construct is coded dichotomously

^b^Please carefully consider the low Cronbach’s alpha (0.39) of this subscale in the present study sample (similar to some previous studies [68, 70]) when interpreting respective results

## Discussion

The aim of the present study was to explore patterns of DEBs reported in the daily lives of young people who were free from a lifetime ED-diagnosis and to examine whether such patterns would be associated with previously established risk factors for EDs. For both females and males, a three-profile solution, including profiles labelled as *no DEBs* (32.2% female, 37.3% male), *high-mixed DEBs* (31.3% female, 27.3% male), and *low-mixed DEBs* (36.5% female, 35.4% male), respectively, was selected. Profiles with elevated DEB-scores (referred to as symptomatic profiles from hereon) were associated with various previously established ED-risk factors, with the female high-mixed-profile showing the largest amount of respective associations, followed by the male high-mixed profile, the female low-mixed profile, and the male low-mixed profile. Within the female symptomatic profiles, associations were found with respect to aspects of psychopathology, eating-and body-related factors, psychological functioning (high-mixed only), interpersonal factors, as well as developmental factors. Male symptomatic profiles were associated with aspects of eating-and body-related factors, interpersonal factors, as well as developmental factors (high-mixed only).

Similarly to the present profile solution, previous latent class analyses have identified DEB-classes characterized by both overeating/binge eating and attempting to limit food intake both in young adult females [26] and young adult males [38]. In a similar manner, a latent class analysis by Stevenson et al. [32] yielded a similar mixed pattern in female and male university students, respectively, characterized by binge eating, restraint, but also other DEBs, such as purging and laxative use. Accordingly, clusters with various mixed DEBs appear to be common in young non-clinical individuals, and the present study has confirmed this even in individuals who are free from lifetime ED-diagnoses. Importantly, in addition to mixed patterns, previous studies have also identified more specific clusters characterized by predominantly one type of DEB [26, 30, 32]. For example, the study by Cain et al. [26] yielded one class endorsing limiting attempts and another class endorsing overeating. By contrast, the present study only yielded two mixed symptomatic profiles distinguished by varying DEB-severities. One might speculate that this may be due to the strictly subclinical nature of the present sample. Previous studies on DEB-patterns in young people have mostly not explicitly excluded individuals with current or lifetime EDs. It is possible that more distinct patterns would emerge if individuals with clinical eating pathologies were included. Nonetheless, it should be noted that, although participants in the high-mixed profile showed elevated levels in all four DEBs examined in the present study, levels of restrained eating were descriptively higher than those of skipping eating, eating large amounts of food, and loss-of-control eating. This resembles previously reported differences in overall prevalence rates of similar DEBs in German adolescents [5] and may indicate that restrained eating may be a particularly common DEB-type in young people with more pronounced, yet subclinical levels, of DEBs.

Moreover, although sex differences in isolated DEBs, including loss-of-control eating and overeating, have been previously reported [89], phenotypes of DEB-patterns appeared to be relatively consistent across both sexes in the present study. This is in line with a previous investigation [32], while other studies have shown sex-related differences in DEB-patterns [26, 38]. Importantly, when considering sex differences, males have been suggested to engage particularly in DEBs focused on achieving muscularity (rather than pursuing a thin body-ideal as often found in females), such as strict dieting practices (e.g., excessive protein consumption) [90]. Accordingly, in the future, when aiming to identify relevant sex differences in patterns of DEBs, it may be beneficial to include additional indicator variables that more specifically reflect symptoms of particular relevance to males.

Further, the sizes of the symptomatic profiles in the present study were quite large, with only approximately 32% of females and 37% of males belonging to the asymptomatic profiles. While similarly high numbers have been previously described for young females [26], others have reported markedly lower numbers [27, 31, 32, 38]. For example, Micali et al. [31], found 87% of 16-year old girls, and 96.5% of 16-year old boys to belong to asymptomatic classes. There may be different reasons for the discrepancies compared to the present study’s findings. On the one hand, differences in indicators used to establish DEB-patterns may offer a possible explanation for the marked variations in asymptomatic and symptomatic group sizes found in different studies. For example, Micali et al. [31] operationalized “weight control behaviors” as purging (self-induced vomiting, using medication to control weight, laxative use) and fasting. Particularly purging behaviors have been shown to be rare in German non-clinical adolescents [5]. Both the study by Cain et al. [26], which also reported high number for symptomatic classes and the present study included less extreme DEBs, such as limiting food intake, which has been shown to be more prevalent in German young non-clinical individuals [5]. On the other hand, one could also speculate that the greater size of symptomatic groups in the present study may result from the assessment of DEBs in daily life, with likely less recall bias compared to assessments using retrospective questionnaires, which could potentially lead to some degree of underreporting of DEBs. Moreover, DEBs within the present study were assessed on a fine-grained scale, ranging from 0 to 100. This likely rather sensitive measurement may have allowed for identifying also milder expressions of DEBs (also reflected in the relatively low DEB-scores in the low-mixed profiles), which may offer another explanation for the rather large symptomatic group sizes in the present study. Generally, when considering the entire female and male analysis samples, low average self-reported severities of DEBs throughout the EMA-period indicated that incidents with more intense DEBs appeared to be infrequent. This could raise the question whether expression of DEBs even reached problematic levels in the present participants.

Nonetheless, validation analyses provided some support for the validity of the symptomatic profiles identified in the present study. Symptomatic profiles were associated with more unfavorable manifestations in a number of correlates (which also have been identified as prospective ED-risk factors in previous studies [e.g., 14, 15, 23, 25, 41–44]) compared to the asymptomatic group in both sexes. Associations were thematically diverse, including aspects of psychopathology, psychological functioning, interpersonal, developmental, and eating-and body-related factors. Generally, this suggests that young individuals of both sexes who engage in DEBs, which may already put them at an increased risk for developing a clinical ED [13–16], show more unfavorable manifestations in various additional risk factors (identified within previous investigations) for ED-development. Moreover, the present findings are in line with previous cross-sectional studies, reporting associations between DEBs and greater affective symptoms [9], difficulties in emotion regulation [91], more pronounced levels of eating to cope with negative emotions [92] lower levels of social support [93], and more pronounced interpersonal problems [94]. Types of correlates associated with symptomatic profiles in the present study also reflect aspects thought to impact the development of EDs from a theoretical perspective according to various theoretical models (for a review, see [24]). As one example for a particularly prominent model, the Transdiagnostic Model of EDs [95] suggests that the over-evaluation of weight, shape, and eating (and their control), difficulties in emotion regulation, interpersonal difficulties, perfectionism and low self-esteem drive eating pathology, including DEBs. While perfectionism was not assessed within the present study and no associations were found between self-esteem and symptomatic profiles, present findings do support associations between DEBs and facets of problems with emotion regulation, interpersonal difficulties (including adverse developmental conditions that may be considered special cases of problematic interpersonal experiences [95]), and over-evaluation of weight, shape, and eating (including engagement in dieting to lose weight), although differences between the different profiles should be taken into consideration.

Moreover, many of the correlates associated with symptomatic profiles in the present sample are also markers of compromised psychological functioning and mental health. Accordingly, awareness regarding DEBs in young people, even if they do not meet diagnostic criteria for a clinical ED, needs to be raised among clinicians. In this context, the focus should be placed on improving screening processes in young people (as suggested by national guidelines [96]), monitoring, implementation of early interventions, as well as targeted prevention. Previous prevention approaches have mostly emphasized eating-and body-related factors (for an overview, see 97) that temporally predate the development of EDs and are associated with a heightened likelihood of their onset, thus making them risk factors in the true sense of word [98, 99]. Based on findings from risk factor research, it has been previously argued that including other aspects, such as interpersonal or affective problems, in prevention programs may appear promising [14]. Findings from the present study support this demand, by underlining the relevance of such thematically more diverse aspects in the context of subclinical eating pathology, albeit only by providing information on correlates (rather than true risk factors) on a cross-sectional level.

However, several limitations must be considered when interpreting the present findings. The relatively short EMA-assessment period, which was selected to reduce participant burden within the comprehensive cohort study assessments, may have resulted in limited reliability in assessing DEBs. A longer assessment period may have yielded potentially more differentiated profiles. While the present study does provide evidence that evaluating DEB-patterns based on repeated reports from everyday life is feasible, future investigations should cover longer assessment periods. In addition, the present LPA only included a limited selection of DEBs as behavioral indicators of eating pathology, while the latter may also encompass further behavioral (e.g., excessive exercise), cognitive (e.g., over-evaluation of weight and shape), and affective (e.g., fear of weight gain) phenomena [48]. Thus, including additional indicators of eating pathology experienced in everyday life in future LPAs may yield more comprehensive and fine-grained phenotypes of subclinical eating pathology and should be considered in future studies.

Another content-related limitation concerns the use of several single-item measures (e.g., self-esteem, body satisfaction, types of attachment styles) within the present study. Single-item measures were chosen in some cases to limit participant burden related to the comprehensive assessments within the BeMIND study, and such measures have frequently demonstrated good validity in previous studies [100, 101]. Nonetheless, assessment of rather complex constructs, such as those examined in the present study, via such single-item measures may limit the conclusions that can be drawn from the present findings and future studies may benefit from applying more sophisticated questionnaires.

Moreover, the present study conducted LPA among a sample relatively heterogeneous in age, including both adolescents and young adults. This may be critical since it has been shown that DEB-patterns may vary at different time points across the course of adolescence [31]. While we did not conduct LPAs separated by age groups in the present study to retain sufficient statistical power, age-stratified analyses are warranted in future investigations. These could not only provide further insights on age-dependent phenotypes, but also age-specific associations with ED-risk factors, which may be particularly important in informing prevention efforts. Further, no sampling weights were applied in the present analyses, which precludes drawing conclusions at a general population level due to the lack of representativeness. Compared to the target population of the BeMIND-study (14–21-years-olds living in Dresden), the present sample contained a higher proportion of females and a lower proportion of older participants [45]. Further, the overall BeMIND study sample includes an overall disproportionately high percentage of individuals with high education [45]. This should be considered when interpreting the present results. Future studies should aim at investigating representative samples or implement appropriate weighing procedures to allow for the generalization of the findings to the general population.

Finally, the present study only investigated cross-sectional associations between DEB-patterns and ED-risk factors previously identified by other studies [e.g., 14, 15, 23, 25, 41–44], precluding any conclusions about whether either symptomatic DEB-profiles or respective risk factors are indeed linked to an increased risk for the development of more serious eating pathology in the present sample. Longitudinal investigations are warranted. Nonetheless, the present study was, to our knowledge, the first study to investigate subclinical DEB-patterns based on daily life-reports from individuals from the general population, providing ecologically valid insights into DEB-patterns shown by young people in their everyday lives.

## Conclusion

The present study suggests that patterns of subclinical DEBs in the daily lives of adolescents tend to be common in both males and females, even in those never diagnosed with an ED. We also found that DEB-patterns are associated with a number of additional risk factors for developing a clinical ED, with more unfavorable manifestations in these risk factors also implying facets of impaired psychological functioning in symptomatic young people. Clinical attention to those who engage in DEBs, even at a non-clinical level, appears warranted.

### Electronic supplementary material

Below is the link to the electronic supplementary material.

Supplementary Material 1.

## Data Availability

The data that support the findings of this study are available from the senior author upon reasonable request.

## Abbreviations

AN: Anorexia Nervosa
ASKU: Short Scale for Measuring General Self-Efficacy Beliefs
BED: Binge-Eating Disorder
BeMIND study: Behavior and Mind Health study
BIC: Bayesian information criterion
BLRT: Bootstrapped likelihood ratio test
BMI-SDS: Body Mass Index standard deviation score
BN: Bulimia Nervosa
DIA-X-5/D-CIDI: Munich Composite International Diagnostic Interview
CROSS-D: Cross-Cutting Dimensional Anxiety Scale
CTQ: Childhood Trauma Questionnaire
DEB(s): Disordered eating behavior(s)
ED(s): Eating disorder(s)
EMA: Ecological momentary assessment
ERSQ: Emotion Regulation Skills Questionnaire
EWI-C: Eating Behaviour and Weight Problems Inventory for Children
LPA: Latent profile analysis
MOPS: Measure of Parental Style
NA: Not applicable
OSLO: OSLO-3-Item-Social-Support Scale
PHQ-9: Patient Health Questionnaire
RQ: Relationship Questionnaire
SABIC: Sample size-adjusted Bayesian information criterion
SISE: Single-Item Self-Esteem Scale
UFED(s): Unspecified Eating Disorder(s)

## References

[1] Pereira RF, Alvarenga M (2007). Disordered eating: identifying, treating, preventing, and differentiating it from eating disorders. Diabetes Spectr.

[2] López-Gil JF, García-Hermoso A, Smith L, Firth J, Trott M, Mesas AE (2023). Global proportion of disordered eating in children and adolescents: a systematic review and meta-analysis. JAMA Pediatr.

[3] Croll J, Neumark-Sztainer D, Story M, Ireland M (2002). Prevalence and risk and protective factors related to disordered eating behaviors among adolescents: relationship to gender and ethnicity. J Adolesc Health.

[4] Neumark-Sztainer D, Wall M, Larson NI, Eisenberg ME, Loth K (2011). Dieting and disordered eating behaviors from adolescence to young adulthood: findings from a 10-year longitudinal study. J Am Diet Assoc.

[5] Schuck K, Munsch S, Schneider S (2018). Body image perceptions and symptoms of disturbed eating behavior among children and adolescents in Germany. Child Adolesc Psychiatry Ment Health.

[6] Nagata JM, Garber AK, Tabler JL, Murray SB, Bibbins-Domingo K (2018). Prevalence and correlates of disordered eating behaviors among young adults with overweight or obesity. J Gen Intern Med.

[7] Sparti C, Santomauro D, Cruwys T, Burgess P, Harris M (2019). Disordered eating among Australian adolescents: prevalence, functioning, and help received. Int J Eat Disord.

[8] Bentley C, Gratwick-Sarll K, Harrison C, Mond J (2015). Sex differences in psychosocial impairment associated with eating disorder features in adolescents: a school-based study. Int J Eat Disord.

[9] Brausch AM, Gutierrez PM (2009). The role of body image and disordered eating as risk factors for depression and suicidal ideation in adolescents. Suicide Life Threat Behav.

[10] Kärkkäinen U, Mustelin L, Raevuori A, Kaprio J, Keski-Rahkonen A (2018). Do disordered eating behaviors have long-term health-related consequences?. Eur Eat Disord Rev J Eat Disord Assoc.

[11] Tabler J, Utz RL (2015). The influence of adolescent eating disorders or disordered eating behaviors on socioeconomic achievement in early adulthood. Int J Eat Disord.

[12] Wade TD, Wilksch SM, Lee C (2012). A longitudinal investigation of the impact of disordered eating on young women’s quality of life. Health Psychol Off J Div Health Psychol Am Psychol Assoc.

[13] Eeden AE, Oldehinkel AJ, Hoeken D, Hoek HW (2021). Risk factors in preadolescent boys and girls for the development of eating pathology in young adulthood. Int J Eat Disord.

[14] Stice E, Gau JM, Rohde P, Shaw H (2017). Risk factors that predict future onset of each DSM-5 eating disorder: predictive specificity in high-risk adolescent females. J Abnorm Psychol.

[15] Stice E, Desjardins CD (2018). Interactions between risk factors in the prediction of onset of eating disorders: exploratory hypothesis generating analyses. Behav Res Ther.

[16] Tanofsky-Kraff M, Shomaker LB, Olsen C, Roza CA, Wolkoff LE, Columbo KM (2011). A prospective study of pediatric loss of control eating and psychological outcomes. J Abnorm Psychol.

[17] Waaddegaard M, Thoning H, Petersson B (2003). Validation of a screening instrument for identifying risk behaviour related to eating disorders. Eur Eat Disord Rev.

[18] Arcelus J, Mitchell AJ, Wales J, Nielsen S (2011). Mortality rates in patients with anorexia nervosa and other eating disorders: a meta-analysis of 36 studies. Arch Gen Psychiatry.

[19] Streatfeild J, Hickson J, Austin SB, Hutcheson R, Kandel JS, Lampert JG (2021). Social and economic cost of eating disorders in the United States: evidence to inform policy action. Int J Eat Disord.

[20] Micali N, Hagberg KW, Petersen I, Treasure JL (2013). The incidence of eating disorders in the UK in 2000–2009: findings from the General Practice Research Database. BMJ Open.

[21] Nagl M, Jacobi C, Paul M, Beesdo-Baum K, Höfler M, Lieb R (2016). Prevalence, incidence, and natural course of anorexia and Bulimia Nervosa among adolescents and young adults. Eur Child Adolesc Psychiatry.

[22] Smink FRE, van Hoeken D, Hoek HW (2012). Epidemiology of eating disorders: incidence, prevalence and mortality rates. Curr Psychiatry Rep.

[23] McClelland J, Robinson L, Potterton R, Mountford V, Schmidt U (2020). Symptom trajectories into eating disorders: a systematic review of longitudinal, nonclinical studies in children/adolescents. Eur Psychiatry.

[24] Pennesi JL, Wade TD (2016). A systematic review of the existing models of disordered eating: do they inform the development of effective interventions?. Clin Psychol Rev.

[25] Ghaderi A (2003). Structural modeling analysis of prospective risk factors for eating disorder. Eat Behav.

[26] Cain AS, Epler AJ, Steinley D, Sher KJ (2010). Stability and change in patterns of concerns related to eating, weight, and shape in young adult women: a latent transition analysis. J Abnorm Psychol.

[27] Calzo JP, Horton NJ, Sonneville KR, Swanson SA, Crosby RD, Micali N (2016). Male eating disorder symptom patterns and health correlates from 13 to 26 years of age. J Am Acad Child Adolesc Psychiatry.

[28] Duncan AE, Bucholz KK, Neuman RJ, Agrawal A, Madden PAF, Heath AC (2007). Clustering of eating disorder symptoms in a general population female twin sample: a latent class analysis. Psychol Med.

[29] Hansson E, Daukantaitė D, Johnsson P (2016). Typical patterns of disordered eating among Swedish adolescents: associations with emotion dysregulation, depression, and self-esteem. J Eat Disord.

[30] Kansi J, Wichstrøm L, Bergman LR (2005). Eating problems and their risk factors: a 7-year longitudinal study of a population sample of Norwegian adolescent girls. J Youth Adolesc.

[31] Micali N, Horton NJ, Crosby RD, Swanson SA, Sonneville KR, Solmi F (2017). Eating disorder behaviours amongst adolescents: investigating classification, persistence and prospective associations with adverse outcomes using latent class models. Eur Child Adolesc Psychiatry.

[32] Stevenson BL, Kwan MY, Dvorak RD, Gordon KH (2018). Empirically derived classes of eating pathology in male and female college students. Eat Disord.

[33] Viborg N, Wångby-Lundh M, Lundh LG, Wallin U, Johnsson P (2018). Disordered eating in a Swedish community sample of adolescent girls: subgroups, stability, and associations with body esteem, deliberate self-harm and other difficulties. J Eat Disord.

[34] Smyth JM, Stone AA (2003). Ecological momentary assessment research in behavioral medicine. J Happiness Stud.

[35] Shiffman S, Stone AA, Hufford MR (2008). Ecological momentary assessment. Annu Rev Clin Psychol.

[36] Goldschmidt AB, Wonderlich SA, Crosby RD, Cao L, Engel SG, Lavender JM (2014). Latent profile analysis of eating episodes in anorexia nervosa. J Psychiatr Res.

[37] Goldschmidt AB, Mason TB, Smith KE, Egbert AH, Engel SG, Haedt-Matt A (2022). Typology of eating episodes in children and adolescents with overweight/obesity. Eat Behav.

[38] Cain AS, Epler AJ, Steinley D, Sher KJ (2012). Concerns related to eating, weight, and shape: typologies and transitions in men during the college years. Int J Eat Disord.

[39] Allen KL, Byrne SM, Forbes D, Oddy WH (2009). Risk factors for full- and partial-syndrome early adolescent eating disorders: a population-based pregnancy cohort study. J Am Acad Child Adolesc Psychiatry.

[40] Bakalar JL, Shank LM, Vannucci A, Radin RM, Tanofsky-Kraff M (2015). Recent advances in developmental and risk factor research on eating disorders. Curr Psychiatry Rep.

[41] Henderson M, Bould H, Flouri E, Harrison A, Lewis G, Lewis G (2021). Association of emotion regulation trajectories in childhood with anorexia nervosa and atypical anorexia nervosa in early adolescence. JAMA Psychiatry.

[42] Johnson JG, Cohen P, Kasen S, Brook JS (2002). Childhood adversities associated with risk for eating disorders or weight problems during adolescence or early adulthood. Am J Psychiatry.

[43] Micali N, Martini MG, Thomas JJ, Eddy KT, Kothari R, Russell E (2017). Lifetime and 12-month prevalence of eating disorders amongst women in mid-life: a population-based study of diagnoses and risk factors. BMC Med.

[44] Stice E (2016). Interactive and mediational etiologic models of eating disorder onset: evidence from prospective studies. Annu Rev Clin Psychol.

[45] Beesdo-Baum K, Voss C, Venz J, Hoyer J, Berwanger J, Kische H (2020). The behavior and Mind Health (BeMIND) study: methods, design and baseline sample characteristics of a cohort study among adolescents and young adults. Int J Methods Psychiatr Res.

[46] Hoyer J, Voss C, Strehle J, Venz J, Pieper L, Wittchen HU (2020). Test-retest reliability of the computer-assisted DIA-X-5 interview for mental disorders. BMC Psychiatry.

[47] Wittchen HU, Lachner G, Wunderlich U, Pfister H (1998). Test-retest reliability of the computerized DSM-IV version of the Munich-Composite International Diagnostic interview (M-CIDI). Soc Psychiatry Psychiatr Epidemiol.

[48] American Psychiatric Association (2013). Diagnostic and statistical manual of mental disorder.

[49] Tomba E, Tecuta L, Crocetti E, Squarcio F, Tomei G (2019). Residual eating disorder symptoms and clinical features in remitted and recovered eating disorder patients: a systematic review with meta-analysis. Int J Eat Disord.

[50] Wade TD, O’Shea A (2015). DSM-5 unspecified feeding and eating disorders in adolescents: what do they look like and are they clinically significant?. Int J Eat Disord.

[51] Heron KE, Scott SB, Sliwinski MJ, Smyth JM (2014). Eating behaviors and negative affect in college women’s everyday lives. Int J Eat Disord.

[52] Fairburn CG, Beglin SJ (1994). Assessment of eating disorders: interview or self-report questionnaire?. Int J Eat Disord.

[53] Peschel SKV, Fürtjes S, Voss C, Sigrist C, Berwanger J, Ollmann TM (2023). Temporal associations between experiential avoidance and disordered eating behaviors in adolescents and young adults: findings from an epidemiological cohort study with ecological momentary assessment. Eat Weight Disord - Stud Anorex Bulim Obes.

[54] Kroenke K, Spitzer RL, Williams JBW (2001). The PHQ-9. J Gen Intern Med.

[55] Löwe B, Spitzer RL, Zipfel S, Herzog W (2002). PHQ-D - manual komplettversion und Kurzform.

[56] Beesdo-Baum K, Klotsche J, Knappe S, Craske MG, Lebeau RT, Hoyer J (2012). Psychometric properties of the dimensional anxiety scales for DSM-V in an unselected sample of German treatment seeking patients. Depress Anxiety.

[57] Lebeau RT, Glenn DE, Hanover LN, Beesdo-Baum K, Wittchen HU, Craske MG (2012). A dimensional approach to measuring anxiety for DSM-5. Int J Methods Psychiatr Res.

[58] Ottova V, Hillebrandt D, Kolip P, Hoffarth K, Bucksch J, Melzer W (2012). Die HBSC-Studie in Deutschland - Studiendesign und Methodik. Gesundheitswesen.

[59] Roberts C, Currie C, Samdal O, Currie D, Smith R, Maes L (2007). Measuring the health and health behaviours of adolescents through cross-national survey research: recent developments in the Health Behaviour in School-aged Children (HBSC) study. J Public Health.

[60] Merikangas KR, Avenevoli S, Costello EJ, Koretz D, Kessler RC (2009). National Comorbidity Survey Replication Adolescent supplement (NCS-A): I. background and measures. J Am Acad Child Adolesc Psychiatry.

[61] Diehl JM (1999). Einstellungen zu Essen und gewicht bei 11- bis 16jährigen adoleszenten [Attitudes to eating and body weight in 11- to 16-year-old adolescents]. Schweiz Med Wochenschr.

[62] Beierlein C, Kemper CJ, Kovaleva A, Rammstedt B (2013). Short scale for measuring general self-efficacy beliefs (ASKU). Methoden Daten Anal.

[63] Robins RW, Hendin HM, Trzesniewski KH (2001). Measuring global self-esteem: construct validation of a single-item measure and the Rosenberg self-esteem scale. Pers Soc Psychol Bull.

[64] Berking M, Znoj H (2008). Entwicklung Und Validierung eines Fragebogens Zur Standardisierten Selbsteinschätzung emotionaler Kompetenzen (SEK-27) [Development and validation of a self-report measure for the assessment of emotion regulation skills (SEK-27)]. Z Für Psychiatr Psychol Psychother.

[65] Bartholomew K, Horowitz LM (1991). Attachment styles among young adults: a test of a four-category model. J Pers Soc Psychol.

[66] Doll J, Mentz M, Witte EH (1995). Zur Theorie Der Vier Bindungsstile: Meßprobleme Und Korrelate dreier integrierter Verhaltenssysteme. [The attachment theory: measurement problems and correlates of three integrated behavioral systems]. Z Für Sozialpsychologie.

[67] Meltzer H (2003). Development of a common instrument for mental health. EUROHIS: developing common instruments for health surveys.

[68] Klinitzke G, Romppel M, Häuser W, Brähler E, Glaesmer H (2012). Die deutsche Version Des Childhood Trauma Questionnaire (CTQ)– psychometrische Eigenschaften in Einer bevölkerungsrepräsentativen Stichprobe. PPmP Psychother Psychosom Med Psychol.

[69] Bernstein DP, Stein JA, Newcomb MD, Walker E, Pogge D, Ahluvalia T (2003). Development and validation of a brief screening version of the Childhood Trauma Questionnaire. Child Abuse Negl.

[70] Gil A, Gama CS, de Jesus DR, Lobato MI, Zimmer M, Belmonte-de-Abreu P (2009). The association of child abuse and neglect with adult disability in schizophrenia and the prominent role of physical neglect. Child Abuse Negl.

[71] Rumpold G, Doering S, Höfer S, Schüßler G (2002). Der Fragebogen dysfunktionaler elterlicher Beziehungsstile (FDEB). Z Für Psychosom Med Psychother.

[72] Parker G, Roussos J, Hadzi-Pavlovic D, Mitchell P, Wilhelm K, Austin MP (1997). The development of a refined measure of dysfunctional parenting and assessment of its relevance in patients with affective disorders. Psychol Med.

[73] Cole TJ, Green PJ (1992). Smoothing reference centile curves: the LMS method and penalized likelihood. Stat Med.

[74] Hemmelmann C, Brose S, Vens M, Hebebrand J, Ziegler A (2010). Perzentilen des Body-Mass-Index auch für 18- bis 80-Jährige? Daten der Nationalen Verzehrsstudie II [Percentiles of body mass index of 18–80-year-old German adults based on data from the Second National Nutrition Survey]. DMW - Dtsch Med Wochenschr.

[75] Kromeyer-Hauschild K, Wabitsch M, Kunze D, Geller F, Geiß HC, Hesse V (2001). Perzentile für den body-mass-index für das Kindes- Und Jugendalter Unter Heranziehung verschiedener deutscher Stichproben. Monatsschr Kinderheilkd.

[76] Posit team. RStudio: Integrated Development Environment for R [Internet]. Boston: Posit Software, PBC; 2023. http://www.posit.co/.

[77] Rosenberg JM, Beymer PN, Anderson DJ, van Lissa Cj, Schmidt JA (2019). tidyLPA: an R package to easily carry out latent profile analysis (LPA) using open-source or commercial software. J Open Source Softw.

[78] Ferguson SL, Moore G, Hull EW (2020). Finding latent groups in observed data: a primer on latent profile analysis in Mplus for applied researchers. Int J Behav Dev.

[79] Morgan GB, Hodge KJ, Baggett AR (2016). Latent profile analysis with nonnormal mixtures. Comput Stat Data Anal.

[80] Spurk D, Hirschi A, Wang M, Valero D, Kauffeld S (2020). Latent profile analysis: a review and how to guide of its application within vocational behavior research. J Vocat Behav.

[81] Schwarz G (1978). Estimating the dimension of a model. Ann Stat.

[82] Sclove SL (1987). Application of model-selection criteria to some problems in multivariate analysis. Psychometrika.

[83] Tein JY, Coxe S, Cham H (2013). Statistical power to detect the correct number of classes in latent profile analysis. Struct Equ Model Multidiscip J.

[84] Celeux G, Soromenho G (1996). An entropy criterion for assessing the number of clusters in a mixture model. J Classif.

[85] McLachlan GJ, Lee SX, Rathnayake SI (2019). Finite mixture models. Annu Rev Stat Its Appl.

[86] StataCorp. Stata Statistical Software: Release 17. College Station. TX: StataCorp LLC. College Station, TX: StataCorp LLC; 2021.

[87] Cohen J (1988). Statistical Power Analysis for the behavioral sciences.

[88] Bender R, Lange S (2001). Adjusting for multiple testing—when and how?. J Clin Epidemiol.

[89] Striegel-Moore RH, Rosselli F, Perrin N, DeBar L, Wilson GT, May A (2009). Gender difference in the prevalence of eating disorder symptoms. Int J Eat Disord.

[90] Lavender JM, Brown TA, Murray SB (2017). Men, muscles, and eating disorders: an overview of traditional and muscularity-oriented disordered eating. Curr Psychiatry Rep.

[91] Trompeter N, Bussey K, Forbes MK, Mond J, Hay P, Cunningham ML (2021). Emotion dysregulation across the span of eating disorder symptoms: findings from a community sample of adolescents. Int J Eat Disord.

[92] Tanofsky-Kraff M, Theim KR, Yanovski SZ, Bassett AM, Burns NP, Ranzenhofer LM (2007). Validation of the emotional eating scale adapted for use in children and adolescents (EES-C). Int J Eat Disord.

[93] Brausch AM, Decker KM (2014). Self-esteem and social support as moderators of depression, body image, and disordered eating for suicidal ideation in adolescents. J Abnorm Child Psychol.

[94] Pine A, Shank LM, Burke NL, Higgins Neyland MK, Schvey NA, Quattlebaum M (2020). An examination of the interpersonal model in adolescent military-dependents at high-risk for adult obesity. Am J Psychother.

[95] Fairburn CG, Cooper Z, Shafran R (2003). Cognitive behaviour therapy for eating disorders: a transdiagnostic theory and treatment. Behav Res Ther.

[96] Herpertz S, Fichter M, Herpertz-Dahlmann B, Hilbert A, Tuschen-Caffier B, Vocks S, et al. (eds). S3-Leitlinie Diagnostik und Behandlung der Essstörungen [Internet]. Berlin, Heidelberg: Springer Berlin Heidelberg; 2019 [cited 2022 Jan 14]. http://link.springer.com/10.1007/978-3-662-59606-7.

[97] Stice E, Johnson S, Turgon R (2019). Eating disorder prevention. Psychiatr Clin North Am.

[98] Jacobi C, Hayward C, de Zwaan M, Kraemer HC, Agras WS (2004). Coming to terms with risk factors for eating disorders: application of risk terminology and suggestions for a general taxonomy. Psychol Bull.

[99] Kraemer HC, Kazdin AE, Offord DR, Kessler RC, Jensen PS, Kupfer DJ (1997). Coming to terms with the terms of risk. Arch Gen Psychiatry.

[100] Ahmad F, Jhajj AK, Stewart DE, Burghardt M, Bierman AS (2014). Single item measures of self-rated mental health: a scoping review. BMC Health Serv Res.

[101] Brailovskaia J, Margraf J (2020). How to measure self-esteem with one item? Validation of the German single-item self-esteem scale (G-SISE). Curr Psychol.

