# Supplementary material for: Subclinical patterns of disordered eating behaviors in the daily life of adolescents and young adults from the general population
Source: Child Adolesc Psychiatry Ment Health. 2024 Jun 6;18:69. doi: 10.1186/s13034-024-00752-w (PMC11157754; doi:10.1186/s13034-024-00752-w)
Supplement: Supplementary file 1 — Supplementary Material 1. [file 13034_2024_752_MOESM1_ESM.docx]

**Supplementary Material**

**Manuscript: Subclinical patterns of disordered eating behaviors in the daily life of adolescents and young adults from the general population**

| **Table S1.** Number of missing values in validation variables in the female subsample | | |
| --- | --- | --- |
| **Variable** | **Missing *n*** | **Missing %** |
| Depressive symptoms PHQ-9 | 4 | 0.74 |
| Anxiety symptoms CROSS-D | 21 | 3.91 |
| Dieting within the past 12 months | 28 | 5.21 |
| Lifetime weight-, shape and eating concerns^a^ | 1 | 0.19 |
| Body satisfaction | 53 | 9.87 |
| Self-esteem SISE | 41 | 7.64 |
| Self-efficacy ASKU | 42 | 7.82 |
| Emotion regulation skills ERSQ | 43 | 8.01 |
| Social support OSLO | 43 | 8.01 |
| Dismissing attachment RQ | 45 | 8.38 |
| Fearful attachment RQ | 45 | 8.38 |
| Preoccupied attachment RQ | 45 | 8.38 |
| Attitude toward the obese EWI-C | 12 | 2.23 |
| Eating as a means of coping with emotional stress EWI-C | 12 | 2.23 |
| Sexual abuse CTQ | 3 | 0.56 |
| Physical neglect CTQ | 3 | 0.56 |
| BMI-SDS | 0 | 0 |
| Maternal over-control MOPS | 50 | 9.31 |
| Maternal abuse MOPS | 50 | 9.31 |
| Maternal indifference MOPS | 50 | 9.31 |
| Paternal over-control MOPS | 81 | 15.08 |
| Paternal abuse MOPS | 81 | 15.08 |
| Paternal indifference MOPS | 81 | 15.08 |
| ASKU, Short Scale for Measuring General Self-Efficacy Beliefs; BMI-SDS, Body Mass Index standard deviation score; CROSS-D, Cross-Cutting Dimensional Anxiety Scale; CTQ, Childhood Trauma Questionnaire; ERSQ, Emotion Regulation Skills Questionnaire; EWI-C, Eating Behaviour and Weight Problems Inventory for Children; MOPS, Measure of Parental Style; n, number; OSLO, OSLO-3-Item-Social-Support Scale; PHQ-9, Patient Health Questionnaire; RQ, Relationship Questionnaire; SISE, Single-Item Self-Esteem Scale.  ^a^ Missings indicate missing values for this particular item within the diagnostic interview. | | |
|  |  |  |
|  |  |  |
|  |  |  |
|  |  |  |
|  |  |  |
|  |  |  |
|  |  |  |

| **Table S2.** Number of missing values in validation variables in the male subsample | | |
| --- | --- | --- |
| **Variable** | **Missing *n*** | **Missing %** |
| Depressive symptoms PHQ-9 | 1 | 0.23 |
| Anxiety symptoms CROSS-D | 1 | 0.23 |
| Dieting within the past 12 months | 37 | 8.62 |
| Lifetime weight-, shape and eating concerns^a^ | 1 | 0.23 |
| Body satisfaction | 58 | 13.52 |
| Self-esteem SISE | 41 | 9.56 |
| Self-efficacy ASKU | 42 | 9.79 |
| Emotion regulation skills ERSQ | 48 | 11.19 |
| Social support OSLO | 48 | 11.19 |
| Dismissing attachment RQ | 51 | 11.89 |
| Fearful attachment RQ | 51 | 11.89 |
| Preoccupied attachment RQ | 51 | 11.89 |
| Attitude toward the obese EWI-C | 15 | 3.5 |
| Eating as a means of coping with emotional stress EWI-C | 15 | 3.5 |
| Sexual abuse CTQ | 4 | 0.93 |
| Physical neglect CTQ | 4 | 0.93 |
| BMI-SDS | 0 | 0 |
| Maternal over-control MOPS | 55 | 12.82 |
| Maternal abuse MOPS | 55 | 12.82 |
| Maternal indifference MOPS | 55 | 12.82 |
| Paternal over-control MOPS | 75 | 17.48 |
| Paternal abuse MOPS | 75 | 17.48 |
| Paternal indifference MOPS | 75 | 17.48 |
| ASKU, Short Scale for Measuring General Self-Efficacy Beliefs; BMI-SDS, Body Mass Index standard deviation score; CROSS-D, Cross-Cutting Dimensional Anxiety Scale; CTQ, Childhood Trauma Questionnaire; ERSQ, Emotion Regulation Skills Questionnaire; EWI-C, Eating Behaviour and Weight Problems Inventory for Children; MOPS, Measure of Parental Style; n, number; OSLO, OSLO-3-Item-Social-Support Scale; PHQ-9, Patient Health Questionnaire; RQ, Relationship Questionnaire; SISE, Single-Item Self-Esteem Scale.  ^a^ Missings indicate missing values for this particular item within the diagnostic interview. | | |
|  |  |  |
|  |  |  |
|  |  |  |
|  |  |  |
|  |  |  |
|  |  |  |
|  |  |  |

**S3. Calculations of differences between included vs excluded individuals**

Differences in sociodemographic characteristics between those included in and excluded from the study’s analyses were examined via linear regression for continuous variables (age, BMI-SDS), logistic regressions for binary outcomes (sex, nationality), and chi-square-tests for outcome variables with multiple categories (social class, education). In the latter case, when the global chi-square-test was significant, pairwise comparisons were conducted to further examine which categories differed between included and excluded participants.

There were some missing values for the sociodemographic variables: *n* = 18 in the included group and *n* = 7 for the excluded group for social class, and *n* = 6 in the excluded group for BMI-SDS. There were no missing values for education, nationality, age, or sex.

**S4. Exclusion of single EMA-assessments**

There were three single EMA-assessments (each from a different participant) in which values for both skipping eating and other types of DEBs were concurrently reported. This should not have occurred due to the filter item described in the methods section in the main text (i.e., participants reporting on skipping eating should not have been presented with any of the other DEB-items within the same EMA-assessment). Assuming that this was due to technical difficulties, all DEB-values from these three assessments were set to missing and not included in the present analyses.


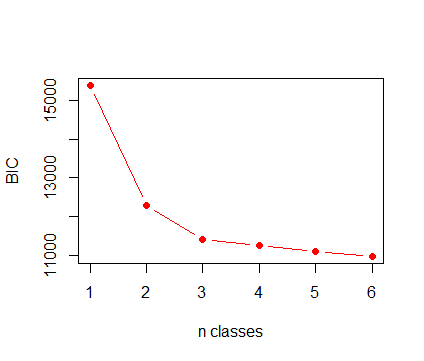


n profiles

BIC

**Fig. S5.** Screeplot of values of the Bayesian information criterion for different profile solutions (females)

*Note.* BIC, Bayesian information criterion; n, number of profiles.


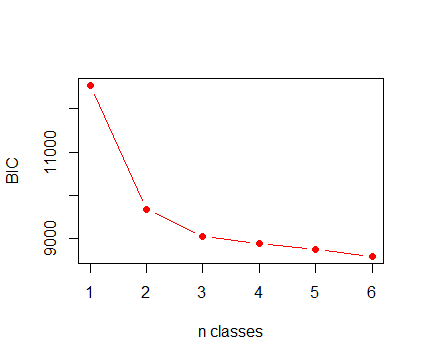


n profiles

BIC

**Fig. S6.** Screeplot of values of the Bayesian information criterion for different profile solutions (males)

*Note.* BIC, Bayesian information criterion; n, number of profiles.

Profile


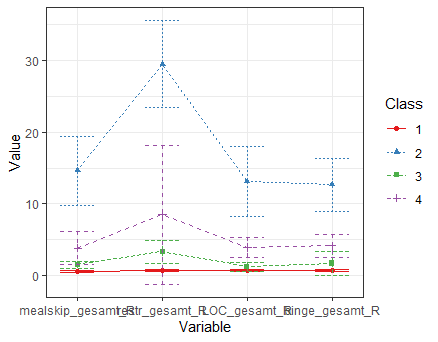


Levels of disordered eating behaviors

Possible range: 0 -100

Skipping eating

Restrained eating

Loss-of-control

eating

Eating large amounts of food

**Fig. S7.** Profile plot for the four-profile solution in females

Profiles

Profile


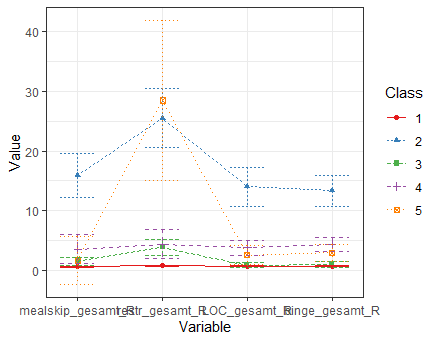


Levels of disordered eating behaviors

Possible range: 0 -100

Skipping eating

Restrained eating

Loss-of-control

eating

Eating large amounts of food

**Fig. S8.** Profile plot for the five-profile solution in females

Profiles

Profile


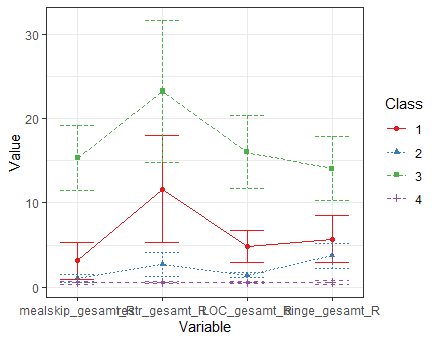


Levels of disordered eating behaviors

Possible range: 0 -100

Skipping eating

Restrained eating

Loss-of-control

eating

Eating large amounts of food

**Fig. S9.** Profile plot for the four-profile solution in males

Profiles

Profile


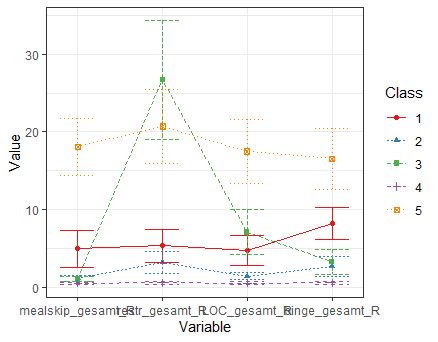


Levels of disordered eating behaviors

Possible range: 0 -100

Skipping eating

Restrained eating

Loss-of-control

eating

Eating large amounts of food

**Fig. S10.** Profile plot for the five-profile solution in males

Profiles
